# Development of a methodology for large-scale production of prions for biological and structural studies

**DOI:** 10.3389/fmolb.2023.1184029

**Published:** 2023-08-10

**Authors:** Luis Concha-Marambio, Fei Wang, Enrique Armijo, Damian Gorski, Frank Ramirez, Andrew Scowcroft, Sandra Pritzkow, Claudio Soto

**Affiliations:** ^1^ Department of Neurology, Mitchell Center for Alzheimer’s Disease and Related Brain Disorders, University of Texas Health Science Center at Houston McGovern Medical School, Houston, TX, United States; ^2^ Amprion Inc., San Diego, CA, United States

**Keywords:** prions, protein misfolding cyclic amplification (PMCA), *in vitro* prion replication, Creutzfelt-Jakob disease, chronic wasting disease

## Abstract

Prion diseases are a group of infectious neurodegenerative diseases produced by the conversion of the normal prion protein (PrP^C^) into the disease-associated form (PrP^Sc^). Extensive evidence indicate that the main or sole component of the infectious agent is PrP^Sc^, which can replicate in affected individuals in the absence of nucleic acids. However, the mechanism of PrP^C^-to-PrP^Sc^ conversion remains elusive, which has been attributed to the lack of sufficient structural information of infectious PrP^Sc^ and a reliable system to study prion replication *in vitro*. In this article we adapted the Protein Misfolding Cyclic Amplification (PMCA) technology for rapid and efficient generation of highly infectious prions in large-scale. Murine prions of the RML strain were efficiently propagated in volumes up to 1,000-fold larger than conventional PMCA. The large-scale PMCA (LS-PMCA) procedure enabled to produce highly infectious prions, which maintain the strain properties of the seed used to begin the reaction. LS-PMCA was shown to work with various species and strains of prions, including mouse RML and 301C strains, hamster Hyper prion, cervid CWD prions, including a rare Norwegian CWD prion, and human CJD prions. We further improved the LS-PMCA into a bioreactor format that can operate under industry-mimicking conditions for continuous and unlimited production of PrP^Sc^ without the need to keep adding brain-derived prions. In our estimation, this bioreactor can produce in 1d an amount of prions equivalent to that present in 25 infected animals at the terminal stage of the disease. Our LS-PMCA technology may provide a valuable tool to produce large quantities of well-defined and homogeneous infectious prions for biological and structural studies.

## Introduction

Prion diseases are rare and invariably fatal neurodegenerative disorders, including Creutzfeldt-Jakob disease (CJD), Chronic Wasting disease (CWD), Bovine Spongiform Encephalopathy (BSE), and Scrapie (Prusiner, 1998). Prion diseases are caused by a proteinaceous infectious agent called prion or PrP^Sc^, an aggregated pathogenic variant of the normal cellular prion protein, termed PrP^C^ (Prusiner, 1998; Soto, 2011). *Prnp*, the gene encoding PrP, is highly conserved among mammalian species (Prusiner et al., 1998; Aguzzi et al., 2008b). Despite this high degree of sequence similarity/homology, PrP^Sc^ from one species is often less efficient or entirely incapable of converting the PrP^C^ of a different species, leading to a prolonged incubation period or absence of clinical signs. This phenomenon, referred to as the species barrier, may be explained by the conformational incompatibility at the interaction interface between host PrP^C^ and exogenous PrP^Sc^ due to the primary structure mismatches among species (Telling, 2000; Hill and Collinge, 2004; Manson and Diack, 2016). Within the same species, prion disease often has different phenotypic manifestations that are associated with distinct prion strains. The strain variants are believed to represent various structural conformations of the same PrP polypeptide (Hill and Collinge, 2004; Aguzzi et al., 2007; Collinge and Clarke, 2007; Morales et al., 2007; Morales, 2017).

The conversion of PrP^C^ into PrP^Sc^ is the primary pathogenic event in the development of prion diseases (Mallucci et al., 2003; White et al., 2003; Aguzzi et al., 2008a). However, the molecular mechanism for PrP^C^-to-PrP^Sc^ conversion is still largely unknown, mostly owing to the lack of sufficient high-resolution structural information of the infectious PrP^Sc^, from natural, relevant prions (Diaz-Espinoza and Soto, 2012). Two structural models have emerged in the past from studies with low resolution techniques: the parallel in-register intermolecular β-sheet (PIRIBS) architecture (Groveman et al., 2014) and the 4-rung β-solenoid model (Vázquez-Fernández et al., 2016). Prions may exist in more than one structural architecture, depending on PrP^C^ primary structure and/or prion strains, as well as the host species. Recent data using cryo-electron microscopy (cryo-EM) and helical reconstruction techniques have provided evidence for the PIRIBS model for at least experimentally cloned strains in rodents (e.g., murine RML and hamster 263K) (Kraus et al., 2021; Hoyt et al., 2022; Manka et al., 2022). Further studies using cryo-EM of more biologically and medically relevant prions are needed to understand the structural basis of infectious prions and prion strains. This information is crucial not only to understand prion propagation, species barrier, and the strain phenomenon, but also for rational development of structure-based therapeutics for these devastating diseases.

Traditionally, wild-type and transgenic animals have been utilized for generating prions through infectivity bioassays, which can be costly and time-consuming. The advent of the Protein Misfolding Cyclic Amplification (PMCA) technique (Saborio et al., 2001; Morales et al., 2012), for the first time enabled the *in vitro* reproduction of the prion replication process, leading to the generation of *bona fide* prions in a test tube after a mere few days (Castilla et al., 2005a; Saá et al., 2006b). When normal animal brain homogenates are used as a source of PrP^C^ to sustain prion replication, PMCA allows the faithful amplification and propagation of prions (Castilla et al., 2005a; Castilla et al., 2008b; Saá et al., 2006b). PMCA has also been developed into a highly specific and sensitive tool to detect prions in bodily fluids (Castilla et al., 2005b; Saá et al., 2006a; Gonzalez-Romero et al., 2008; Moda et al., 2014; Concha-Marambio et al., 2016). Multiple lines of evidence from different labs have shown that PMCA amplified prions preserve the original strain properties, namely the biochemical, biophysical, and biological activities, of the seed prions (Castilla et al., 2008b; Green et al., 2008; Shikiya et al., 2010; Bucalossi et al., 2011; Saa et al., 2012). A significant amount of knowledge and insight have been gained from PMCA studies of prion propagation, species barrier, and strain properties (Castilla et al., 2005a; Castilla et al., 2008a; Castilla et al., 2008b; Green et al., 2008; Meyerett et al., 2008). In this respect, PMCA can serve as a surrogate for animal assays to generate and propagate *bona fide* prions. However, regular PMCA has been standardized for faithful propagation of prions in PCR tubes with a typical reaction volume of 100 µL (Morales et al., 2012). Another limitation is the use of brain homogenates as a source of PrP^C^ in PMCA studies. Several studies have been done to utilize recombinant PrP in PMCA as a more convenient source of PrP^C^ (Kim et al., 2010; Makarava et al., 2010; Wang et al., 2010; Schmidt et al., 2015). However, most of the attempts reported so far have yielded a lower infectivity titer, and often the inability to faithfully propagate PrP^Sc^ strain properties (Schmidt et al., 2015).

In this study, we report the adaptation of PMCA to a large-scale format and the development of a PMCA-based bioreactor to produce high quantities of *bona fide* prions for future biological and structural studies.

## Materials and Methods

### Ethics statement

This study was carried out in strict accordance with the recommendations in the Guide for the Care and Use of Laboratory Animals of the National Institutes of Health. The animal protocol was approved by the Institutional Animal Care and Use Committee of the University of Texas Health Science Center at Houston.

### PMCA substrates

As substrate for the PMCA reaction we use the appropriate brain homogenate (see below) as a source of PrP^C^. The brain was collected and processed as previously described (Morales et al., 2012), except with the modifications outlined below for deer and human substrates. *Mouse PrP (for RML and 301C propagation)*: 10% brain homogenate was prepared with perfused (1X PBS supplemented with 5 mM EDTA) wild-type mouse whole brains in conversion buffer (1X PBS supplemented with 1% Triton X-100, 150 mM NaCl, and Complete, EDTA-free protease inhibitor). Large debris was removed by brief centrifugation at 810 x g at 4°C for 1 min. The supernatant was aliquoted, snap-frozen in liquid nitrogen, and stored at −80°C until use.

*Hamster PrP (for Hyper propagation)*: 10% BH was prepared from Golden Syrian Hamster brains as described above.

*Deer PrP (for both North America and Norwegian CWD propagation)*: 10% BH was prepared from Tg Gt226Q mouse brains (kindly provided by Dr. Glenn Telling) as above. Tg Gt226Q is a knock-in mice that express the sequence of the deer prion protein. Directly before PMCA, brain homogenate was thawed and supplemented with 12 mM EDTA, 0.05% Digitonin, and 100 μg/mL heparin.

*Human PrP (for both vCJD and sCJD propagation)*: 10% BH was prepared from Tg 6815 (129M) or Tg 7826 (129V) mouse brains (kindly provided by Dr. Glenn Telling) as above. These transgenic mice overexpress the human prion protein gene with methionine or valine at position 129. Directly before PMCA, brain homogenate was thawed and supplemented with 12 mM EDTA, 0.05% Digitonin, and 100 μg/mL heparin for amplifying vCJD. For sCJD PMCA, additional 0.01% sodium tripolyphosphate (STPP) was included. EDTA, digitonin, heparin and STPP were added to increase PMCA efficiency, as identified in our previous publications.

### Standard PMCA procedure

Regular PMCA, was performed as described previously (Morales et al., 2012). Briefly, thin PCR tubes (Eppendorf, Cat. No. 951010022) were loaded into the holder of a QSonica Q700 sonicator equipped with a titanium horn. 220–250 mL of water was poured into the holder in every experiment. Each sonication cycle comprised 20 s of sonication at an amplitude of 13 and 29 min 40 s of incubation. The horn and converter of the sonicator were placed in a 32°C incubator. Each PMCA round lasted 48 h. Reactions containing three 3/32 inch PTFE (polytetrafluoroethylene) beads were performed to compare the efficiency between standard PMCA and LS-PMCA.

### LS-PMCA

#### First generation—Spherical containers

**FIGURE 1:**
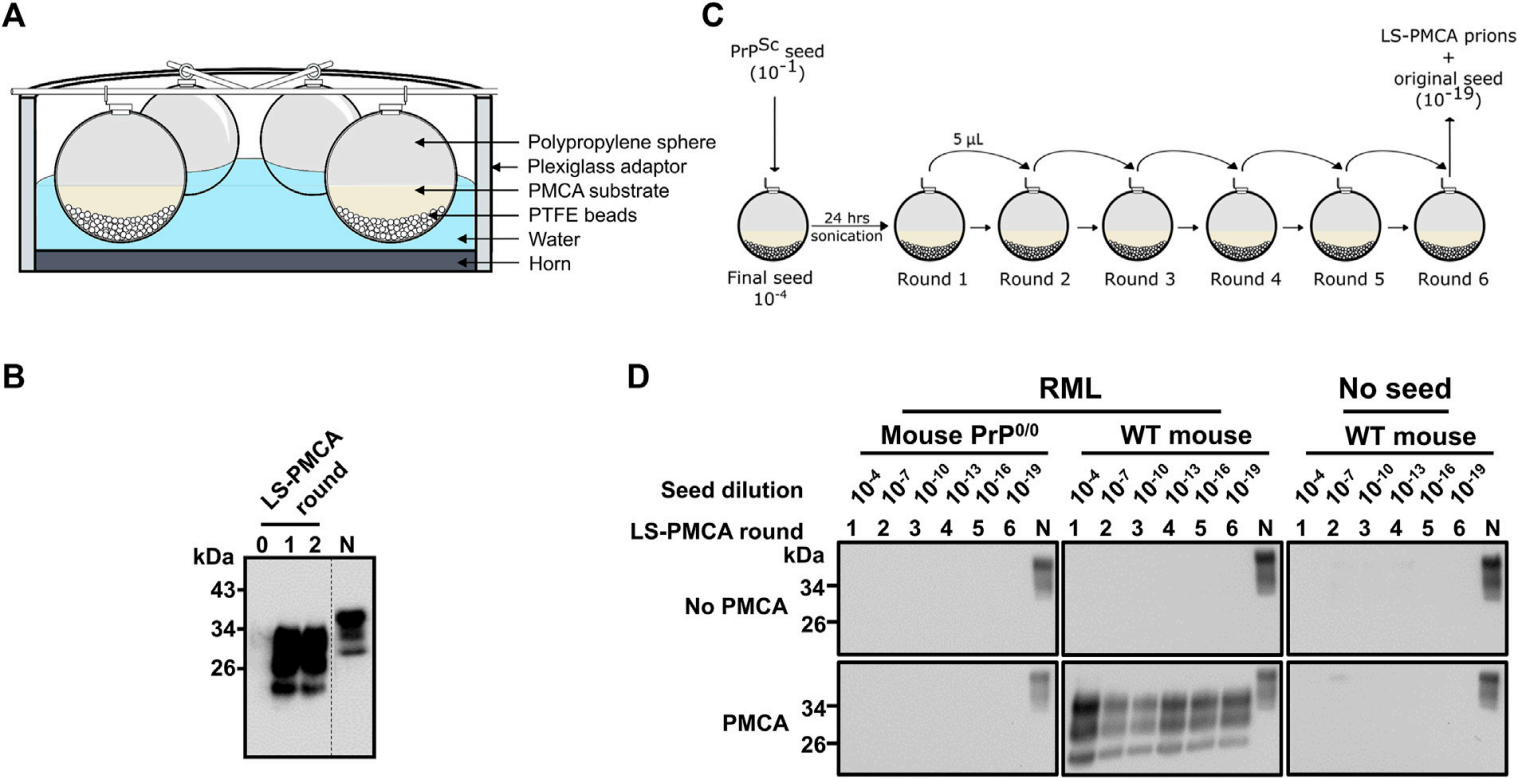
Large-scale PMCA for the *in vitro* production of infectious prions. **(A)** Diagram of the experimental setup. 6 polypropylene spheres per sonicator were loaded with 5 mL of normal mouse brain homogenate (BH) and seeded with 10^–4^ final dilution of RML BH. **(B)** Amplification of RML prions at days 0, 1, and 2 of programmed sonication. PMCA samples at different time points were treated with 25 μg/ml PK and analyzed by SDS-PAGE and western blotting using the monoclonal antibody 6D11. Dotted line denotes blot splicing to remove irrelevant lanes, but all the samples in this blot were ran in the same gel. **(C)** Diagram of the serial LS-PMCA experiment. A polypropylene sphere containing 5 mL of normal mouse BH was seeded with 5 µL of RML BH (final dilution of 10^–4^ in the reaction). After 24 h of programmed sonication (1 round of LS-PMCA), 5 µL of the reaction was used to seed a new sphere containing fresh normal mouse BH. Hence, the brain-derived RML prion was diluted additional 1,000 times (10^–7^ after the 2nd round of PMCA). Six rounds were performed, reaching a final dilution of the brain-derived RML prion of 10^–19^. **(D)** Western blot analysis of each round of LS-PMCA. Samples before (No PMCA) and after sonication (PMCA) were analyzed for each round. Two types of BH were used: normal mouse BH (WT mouse) and PrP knockout mice (Mouse PrP^0/0^), in the presence or absence of the RML BH. N is 5 µL of normal mouse BH used as migration control.

Hollow polypropylene spheres from Product Components Corp (1 ½” diameter, Cat. No. P/N J57004) were used as the vessel for the first-generation LS-PMCA. A small hole of the exact size of an Eppendorf tube was drilled on the spheres and the cup of a tube was used to seal it. LS-PMCA containing 5 mL PMCA substrate and 5 µL PMCA seed was supplemented with 100 PTFE beads of 3/32 inch diameter (Hoover precision products) and cycles of amplification were carried out with a QSonica Q700 sonicator equipped with a titanium horn at an amplitude of 35. A Plexiglas adaptor was used to ensure enough water was added to reach the same level as the reaction mixture inside the spheres (Figure 1A). Each sonication cycle comprised 20 s of sonication at the amplitude of 20 and 29 min 40 s of incubation. The horn and converter of the sonicator were placed in a 32°C incubator. Each LS-PMCA round lasted 24 h (48 cycles).

#### Second generation—Cup containers

*20 mL cup*: Polypropylene cups from Fisher Scientific (Cat. No. 23-032059) were used as a vessel for the second-generation LS-PMCA (20 mL capacity). The cups were rinsed thoroughly with Nanopure water and dried. To start a 20 mL capacity cup LS-PMCA, 5 mL PMCA substrate, 5 µL PMCA seed, and 100 PTFE beads of 3/32 inch diameter were added to the cup, which was then placed on a 3D printed cup holder that fits the Plexiglas adaptor. The mixture was subjected to 20 s of sonication with the amplitude of 35 and 29 min 40 s of incubation. Small amounts of LS-PMCA products at different time points were removed from the cup for characterization purposes. At the end of the initial experiment, the LS-PMCA product was removed from the bioreactor cup until no liquid can be suctioned up into the pipette tips. The initial LS-PMCA product was labeled as batch number one. For the second batch product, only 5 mL of the substrate and no additional prion seed were added to the same bioreactor cup and subjected to LS-PMCA. Consecutive batches of prions were generated in a similar way (Figure 5A).

*500* *mL cup*: Polypropylene cups from Thermo Fisher (Cat. No. 2118-0016) were used as a vessel for the second-generation LS-PMCA (500 mL capacity). The containers were rinsed thoroughly with Nanopure water and dried. To start a 500 mL capacity cup LS-PMCA, 100 mL PMCA substrate, 100 µL PMCA seed, and 1,700 PTFE beads of 3/32 inch diameter were added to the cup, which was then placed on an in-house styrofoam cup holder that fits the Plexiglas adaptor, and subjected to PMCA (20 s of sonication with the amplitude of 35 and 29 min 40 s of incubation). Small amounts of LS-PMCA products at different time points were removed from the cup for characterization purposes.

#### Bioassay

Four-week-old female C57BL/6J mice were used for the bioassay. For intracerebral inoculations, animals were anesthetized with isoflurane and received a stereotaxic injection of 5 µL inoculum into the hippocampal zone following sterile surgical techniques. The animals were monitored weekly and euthanized when clinical signs progressed to advanced stages.

#### Proteinase K (PK) digestion and western blotting

PMCA products and standard prion-laden brain homogenates were incubated with PK for 1h at 37° with agitation. Mouse and hamster prions were incubated with 50 μg/mL PK for 1 h at 37° unless stated otherwise, human and deer prions with 100 μg/mL for the same time and at the same temperature. PK digestions were stopped by the addition of loading sample buffer and boiling for 10 min at 100°C. Proteins were separated by SDS-PAGE and then transferred to 0.45 µm nitrocellulose membranes, which were blocked with 10% dry milk for 1 h at RT and then probed with monoclonal antibody 6D11 unless stated otherwise.

#### Histology

Brain samples were fixed in Carnoy solution (60% ethanol, 30% chloroform, and 10% acetic acid), dehydrated, and embedded in paraffin. 7 μm tissue slices were stained with hematoxylin-eosin (H&E) or immuno-stained with monoclonal antibody to PrP (6H4, 1:1000; Prionics^®^). For staining PrP^Sc^ C-terminal fragment produced after PK digestion (PrPres), slides were treated with 6% hydrogen peroxide for 20 min and subsequently subjected to 10 μg/mL PK for 5 min and 3 M GndCl for 20 min. Slides were incubated overnight using the 6H4 antibody (Prionics^®^) and non-specific binding was prevented using the Dako ARK^®^ (Animal Research Kit) following the manufacturer’s recommendations. Immunostaining was developed using HRP-conjugated streptavidin and visualized with DAB as a chromogen. Tissues were counterstained with hematoxylin for 30 s and rinsed in tap water for 10 min. Later, slides were dehydrated in ≥95% ethanol, cleared with xylene, and mounted with a resinous mounting medium. Sections were examined under a bright field/epifluorescent DMI6000B Leica^®^ microscope. Vacuolation profiles were determined on H&E-stained sections, by scoring the spongiform degeneration changes in the nine standard grey matter areas.

#### Biosafety precautions

Since prions are infectious, all studies with rodent or deer prions were done in a dedicated BSL2 facility and studies with human prions were done in a BSL3 facility dedicated exclusively to work with human prions. All waste was decontaminated and incinerated as described previously (Morales et al., 2012). Procedures to work with prion infectious material were approved by the Biosafety Committee of the University of Texas Health Science Center at Houston.

## Results

### Implementation of large-scale protein misfolding cyclic amplification (LS-PMCA)

The standard PMCA reactions take place in PCR tubes with a typical reaction volume of 100 µL (Morales et al., 2012). To assess if prions can be amplified in much larger reaction volumes, spherical containers were first tested as LS-PMCA vessels (Figure 1A). In the initial setup, 5 mL normal mouse brain homogenate (BH) was mixed with 5 µL of 10% RML BH and subjected to 2 rounds of PMCA. The result showed a clear amplification of PrP^Sc^ in this setting (Figure 1B). Interestingly, only 1 day of PMCA cycling (i.e., one round of LS-PMCA, see Materials and Methods) was enough to achieve maximum conversion, as the second round of PMCA did not produce a higher amount of PrPres (Figure 1B). Before PMCA, no PrPres reactivity was observed by western blot (see round 0 in Figure 1B), indicating that the PK resistant bands observed after 1 and 2 rounds of LS-PMCA were produced by *in vitro* amplification since the initial inoculum was diluted below the detection limit of western blot (10^–4^). The LS-PMCA amplification efficiency is comparable to that of the standard PMCA and the electrophoretic pattern of the PK-digested LS-PMCA products is identical to that of the PK-resistant products obtained in standard PMCA (Supplementary Figure S1A).

To evaluate the consistency of LS-PMCA amplification, we set up a series of LS-PMCA starting with 10^–1^ RML BH, which was diluted to 10^–4^ by seeding the 5 mL of 10% mouse BH substrate in the first round of LS-PMCA. The original RML BH was diluted 1,000 more times in each subsequent round, reaching a 10^–19^ dilution after 6 rounds of LS-PMCA (Figure 1C). We found PrPres in the LS-PMCA products of all 6 rounds, demonstrating the robust and indefinite replication of RML prions in the LS-PMCA setting (Figure 1D, RML, Wt mouse). To demonstrate the absence of *de novo* formation of prions and rule out potential cross-contamination, the same substrate in the absence of RML seed was subjected to LS-PMCA. All rounds yielded no detection of PrPres, validating that the production of PrPres resulted from a seeded propagation (Figure 1D, No seed, Wt mouse). Finally, we conducted the same experiment using brain homogenate from PrP knockout mice (PrP^0/0^) as substrate to prove that host PrP^C^ is necessary for PrP^Sc^ production (Figure 1D, RML, Mouse PrP^0/0^). In addition, all conditions used in this experiment were also analyzed by western blot in the absence of PMCA cycles. No PrPres was detected in any round of any condition, confirming that prions generated by LS-PMCA are the products of the cyclic fragmentation and elongation of PrP^Sc^, as by standard PMCA (Figure 1D, No PMCA).

### Generation of *bona fide* infectious prions by LS-PMCA

**FIGURE 2:**
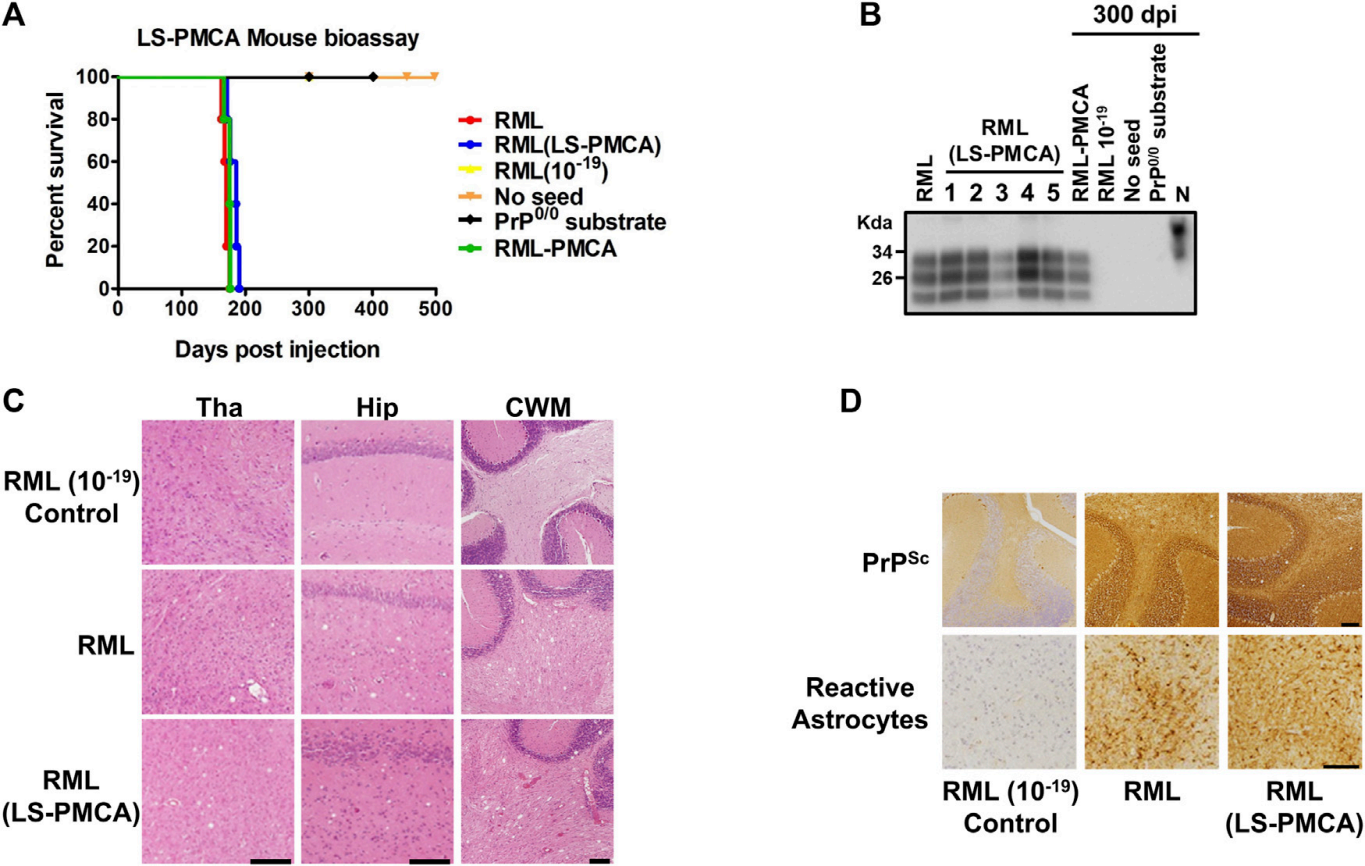
Bioassay of LS-PMCA generated prions. **(A)** C57BL/6J mice were intracerebrally injected with brain-derived RML and *in vitro* generated RML by either LS-PMCA or standard PMCA. As controls, RML BH at the dilution of 10^–19^, the unseeded PMCA product (with normal mouse BH as substrate), and the seeded PMCA product (with PrP knockout mouse BH as substrate) were injected as well. The graph shows the survival time, measured as the number of days it took for animals to reach the terminal stage of the disease and to be humanely euthanized. Animals which did not show any clinical symptoms, were kept for 500 days. **(B)**. Analyses of PrPres from injected animals. Symptomatic animals were sacrificed at terminal stage (RML, RML (LS-PMCA), and RML-PMCA). Of the control groups, one animal was sacrificed at 300 dpi (RML 10^–19^, No Seed, and PrP^0/0^ substrate). The brains were collected, homogenized, and digested with 50 μg/mL PK. N is 5 µL of mouse BH used as migration control. **(C)** Vacuolation analyses after H&E staining. Representative images from different brain regions from control, RML, and LS-PMCA RML animals. Tha: Thalamus, Hip: Hippocampus, CWM: Cerebellar White Matter. Scale bar: 100 µm. **(D)** Immunohistochemical analyses. Representative images of PrP^Sc^ (detected by 6H4 antibody) deposition in the cerebellum from control, RML, and LS-PMCA RML animals. Representative images of reactive astrocytes (detected by anti-GFAP antibody) in the Thalamus from control, RML, and LS-PMCA RML animals. Left panels: control; middle panels: RML; right panels: LS-PMCA RML. Scale bar: 100 µm.

Although it has been well established that regular PMCA products faithfully retain the key properties of the original prion seeds, including both infectivity and strain characteristics (Castilla et al., 2005a; Castilla et al., 2008b), we performed bioassay to confirm the faithful PrP^Sc^ propagation of the RML strain by LS-PMCA. Wild-type C57BL/6J mice were inoculated with the LS-PMCA product of the 6th round, which theoretically contained 10^–19^ original RML seed. Remarkably, the survival curve of the mice inoculated with LS-PMCA product is indistinguishable from those of the mice infected with either brain-derived RML or the products of standard PMCA (Figure 2A; Supplementary Table S1). This suggests that LS-PMCA produces a concentration of infectious prions similar to a 10% brain homogenate from an infected animal at the terminal stage of the disease. As expected, inoculation of brain-derived RML diluted to 10^–19^ did not cause disease in any of the 5 injected animals, demonstrating that the 10^–19^ dilution should not contain any brain-derived prion infectious material. This indicates that the disease caused in samples amplified by LS-PMCA and standard PMCA was produced by the *in vitro* formation of prions by PMCA. Additionally, we injected wild-type C57BL/6J mice with the round 6 LS-PMCA product generated in a non-seeded reaction and with the round 6 LS-PMCA product generated in an RML-seeded reaction using PrP^0/0^ BH as substrate. None of these materials caused prion disease in any of the injected animals even after 500 dpi (Figure 2A).

Half of the brain tissues from these mice were homogenized for PK-digestion and western blot analyses (Figure 2B). All 5 animals injected with the material generated by LS-PMCA displayed PrPres with the electrophoretic patterns of classical RML strain, in terms of the mobility and glycosylation pattern (Figure 2B), which were also observed in animals injected with RML generated by standard PMCA. After 300 dpi, one animal from each of the remaining groups was evaluated and no PrPres was detected in the animals injected with 10^–19^ brain-derived RML, the 6th round products with no seed or with PrP^0/0^ substrate (Figure 2B). The other half of the brains were used to analyze neuropathological changes. While the control mice showed no vacuolation, LS-PMCA RML injected mice developed spongiosis in multiple brain regions to an extent and distribution similar to RML BH injected mice (Figure 2C). Likewise, immunohistochemical staining revealed similar astrogliosis and PrP^Sc^ deposition patterns in both RML BH and LS-PMCA RML inoculated mice (Figure 2D). Taken together, our *in vitro* and *in vivo* data supports that the LS-PMCA setting produces highly infectious prions retaining the strain properties of the original prions.

### Second-generation LS-PMCA

**FIGURE 3:**
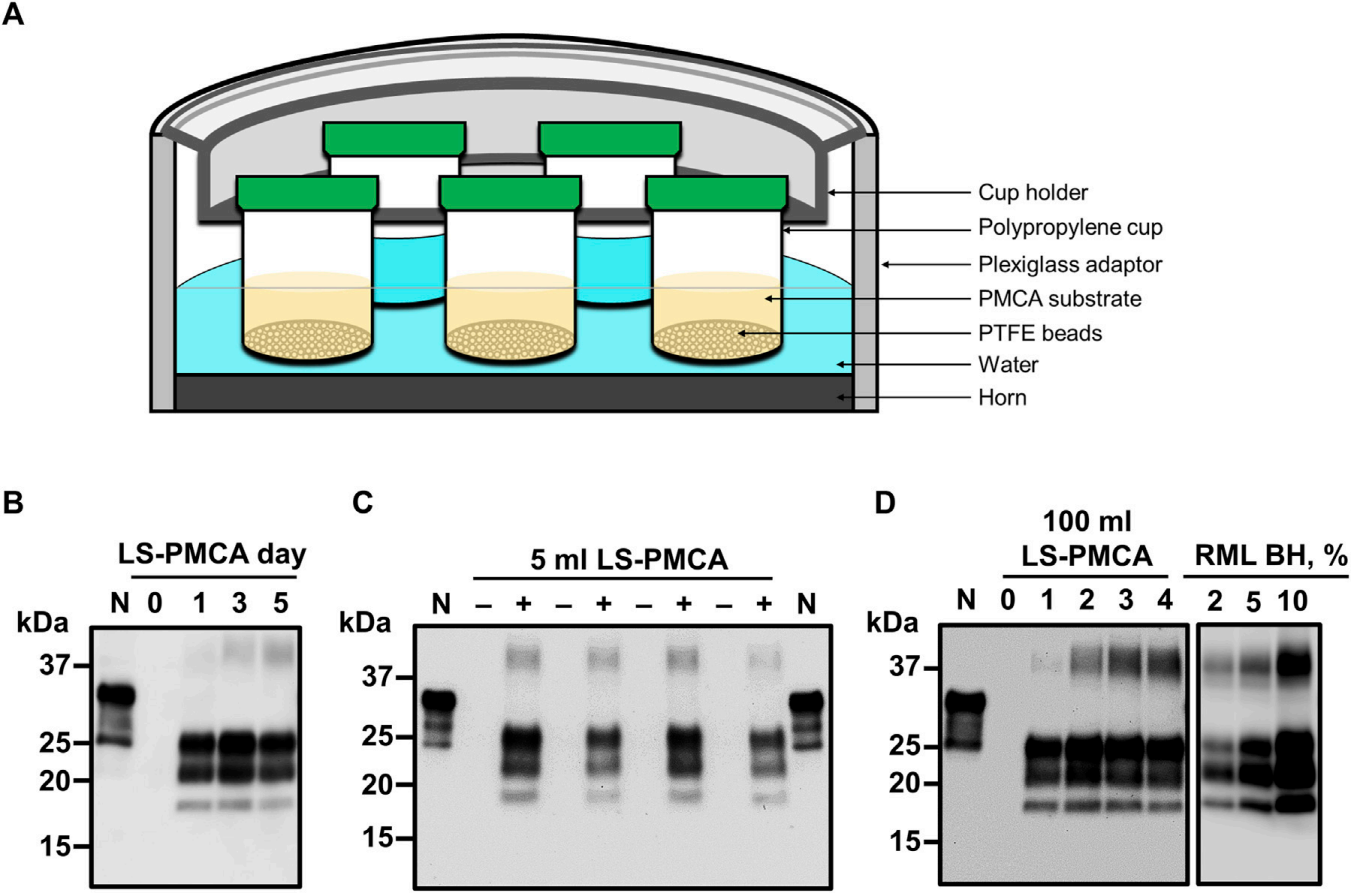
Second-generation LS-PMCA. **(A)** Diagram of the experimental setup. Polypropylene cups were loaded with 5 mL of normal mouse BH and 5 µL of RML BH. **(B)** Amplification of RML prions at days 0, 1, 3, and 5 of programmed sonication in a 20 mL cup (reaction volume: 5 mL). **(C)** Amplification of RML prions in different 20 mL cups (reaction volume: 5 mL) from 4 separate experiments. **(D)** Amplification of RML prions at days 0, 1, 2, 3, and 4 of programmed sonication in a 500 mL cup (reaction volume: 100 mL). PMCA samples at different time points were treated with 50 μg/ml PK for 1 h and analyzed by SDS-PAGE and western blotting using the monoclonal antibody 6D11. For comparison, different dilutions of RML brain homogenate (2%, 5% and 10%) after PK treatment were included in the right panel.

Next, we aimed to expand the reaction volume of LS-PMCA to increase yield. For the ease of handling the increased volume of substrates and number of beads, we set to test wide-mouth cups as the second-generation container for LS-PMCA (see details in Materials and Methods). We started with a 20 mL capacity cup to conserve the substrate, seed, and beads (Figure 3A). In the same LS-PMCA setting, 5 mL of substrate, 5 µL of RML seed, and 100 beads were added to the cup and subjected to cycles of sonication and incubation. At days 0, 1, 3, and 5, aliquots of 10 µL of PMCA product were removed from the container and subjected to PK-digestion, SDS-PAGE separation, and immunoblotting analyses (Figure 3B). Compared to day 0 (prior to PMCA), PrPres was detected after 1, 3, and 5 days of PMCA. As with the first-generation LS-PMCA, the propagation of PrPres reached a plateau after 1 day of LS-PMCA, indicating that the second-generation LS-PMCA amplification efficiency is comparable to that of the first-generation LS-PMCA. The amplification efficiency of LS-PMCA was compared side-by-side with standard PMCA. The product of the reaction in both cases contains an indistinguishable amount of PrPres detected by western blots (Supplementary Figure S1A). The robustness of the second-generation LS-PMCA was validated by repeating the propagations in multiple cup containers in separate experiments (Figure 3C). Encouraged by the results in the 20 mL container, we furthered the second-generation LS-PMCA into a 500 mL capacity container. Without a change in the sonication and incubation parameters, PrPres was generated as efficiently in a 100 mL reaction (500 mL container) as in the 5 mL reaction (20 mL container) (Figures 3B, D). To estimate the quantity of PrP^Sc^ produced by LS-PMCA compared with brain of an infected mice, we performed western blot after PK-digestion. Our results suggest that the concentration of PrP^Sc^ produced by LS-PMCA was equivalent to that of at least a 5% brain homogenate from a sick animal (Figure 3D). This supports the data obtained by infectivity bioassay (Figure 2A) showing that the material produced by LS-PMCA was equivalent to a 10% infected brain homogenate. This conclusion is further supported by measuring the amount of PrP^Sc^ produced after LS-PMCA and compared with the amount of PrP^C^ present in the reaction substrate. The result of this experiment showed that virtually all PrP^C^ was successfully converted to PrP^Sc^ by LS-PMCA (Supplementary Figure S1B). In the case of mice, 1 animal brain can produce 4 ml of 10% homogenate. Thus, LS-PMCA done in a 100 ml solution, contain a quantity of PrP^Sc^ equivalent to 25 terminally ill mice. Furthermore, to produce this amount of prions via mouse bioassay, it is necessary to wait typically around 150–200 days. In comparison, LS-PMCA produces this quantity of PrP^Sc^ in only 1 day.

### Amplification of various natural prions with LS-PMCA

**FIGURE 4:**
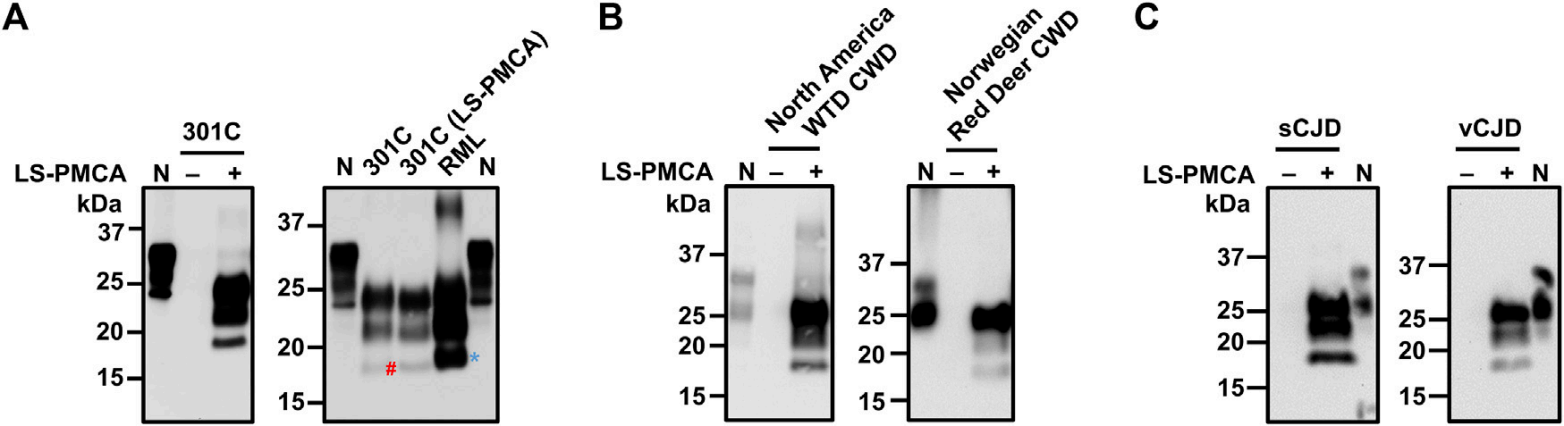
Amplification of different prions by LS-PMCA. **(A)** Mouse prion 301C was amplified by LS-PMCA (left panel). Electrophoresis mobility of LS-PMCA amplified 301C prion was compared to those of native mouse 301C and RML prions (right panel). The blue asterisk and red pound sign mark the unglyscosylated band of PrPres in RML and 301C, respectively. **(B)** Both North American White Tail Deer (WTD) CWD prion and a rare Norwegian CWD prion found in a Red Deer were amplified by LS-PMCA using transgenic mouse BH expressing deer PrP. Anti-PrP antibody 6H4 was used for detecting deer PrP. **(C)** sCJD (VV2) and vCJD prions were amplified by LS-PMCA using transgenic mouse brain homogenate containing V129 and M129 Human PrP, respectively.

To evaluate the capabilities of LS-PMCA to propagate prions from different species and/or different strains of the same host species, we first performed LS-PMCA to amplify another mouse-adapted prion, 301C. LS-PMCA of different prions was carried out in the 20 mL cups containing 5 mL substrates. In the same setting, LS-PMCA was able to efficiently generate PrP^Sc^ in the reaction containing wild-type mouse BH substrate and 301C seed (Figure 4A, left panel). LS-PMCA propagated 301C prions migrated on SDS-PAGE similarly as the original 301C seed after protease digestion, with the 301C prion specific un-glycosylated PK-resistant band running faster than that of RML prion (Figure 4A, right panel). We also found that LS-PMCA was able to efficiently propagate hamster Hyper prions over 6 rounds of amplification (Supplementary Figure S2), similar to RML prion (Figure 1).

We further tested whether LS-PMCA was able to amplify certain more relevant prions, namely cervid CWD and human prions. Using BH from transgenic mice expressing deer PrP as substrate, we were able to amplify North American CWD prion in LS-PMCA (Figure 4B, left panel). Moreover, LS-PMCA was capable of propagating a novel Norwegian CWD identified in a wild Red Deer (Vikøren et al., 2019) (Figure 4B, right panel). Lastly, two human prions, a subtype (VV2) of sporadic Creutzfeldt-Jakob disease (sCJD) and the variant CJD, could be efficiently amplified by LS-PMCA using BH from transgenic mice overexpressing human PrP as substrate as shown in Figure 4C.

## Implementation of a LS-PMCA-based bioreactor for prion production

**FIGURE 5:**
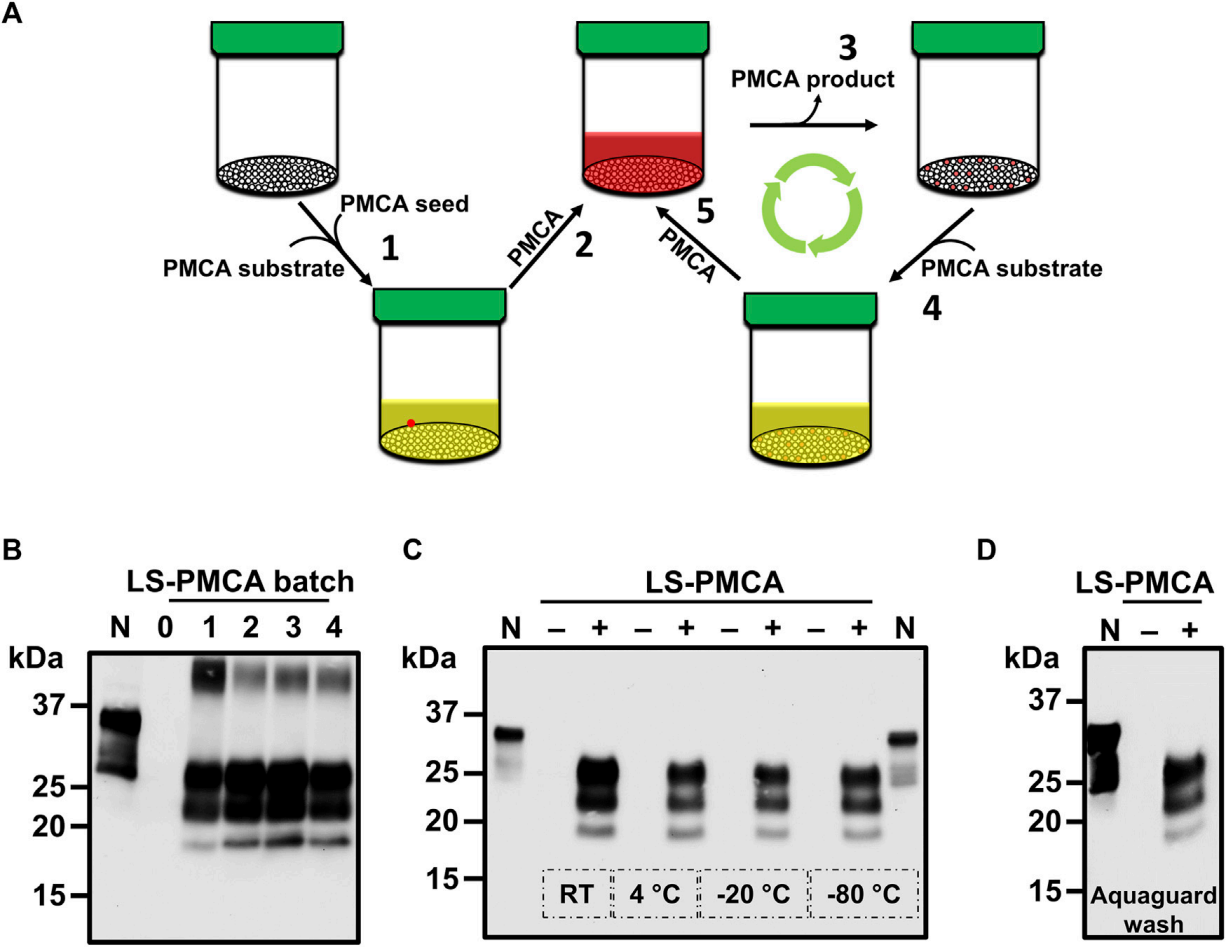
Development of LS-PMCA bioreactor. **(A)** Illustration of developing LS-PMCA to a Bioreactor format. Step 1. The polypropylene cup is loaded with a large amount of PMCA substrate and a minute amount of prion seed. Step 2. After PMCA, the substrate is converted and the prion is amplified. Step 3. The PMCA amplified prion is removed from the cup and a trace amount of prion is left in the cup, most likely on surfaces of beads and walls. Step 4. A large amount of fresh PMCA substrate is added to the same cup. Step 5. PMCA converts substrates and amplifies prions. Repeating Steps 3–5 can be done as many times as needed to replicated prions indefinitely. In this Bioreactor, the original prion seed is only required for the first batch. For the subsequent batches, no extra original prion infectious material is added. **(B)** RML prion was amplified in the Bioreactor. After PMCA, the 1st batch of LS-PMCA amplified RML prion was removed and another fresh 5 mL normal mouse BH was added to the same Bioreactor. Batches 2, 3, and 4 of RML prion were then amplified in the same Bioreactor. **(C)** To analyze the stability of prions kept in the bioreactor, after amplification and removal of the first batches of RML prions, the cups were placed either at room temperature (RT), 4°C, −20°C, or −80°C for 1 month, then re-activated to amplify the second batches of RML prion. The figure shows the presence of PrP^Sc^ in the second batch. Note: no extra seed was added. **(D)** To evaluate whether the bioreactor will be affected by treatment with anti-microbial agents, a bioreactor was washed twice with Aquagauard solution (5 mL wash each time) after amplification and removal of a first batch of RML prion. The cup was left at RT for 2 weeks, then re-activated to amplify another batch of RML prion. The figure shows the results obtained in the second batch of amplification. Note: no extra seed was added.

Next, we wanted to establish a platform for the large-scale production of infectious prions for biological and structural studies. To mimic the industrial large-scale production of enzymes or pharmaceutical substances, we sought out to develop the LS-PMCA as a Biological Reactor, or Bioreactor, in order to maximize the yield while simultaneously minimizing the cost. Taking advantage of the ultrasensitive detection and amplification ability of PMCA (Castilla et al., 2005a; Morales et al., 2012), we tested the possibility of amplifying one specific prion strain in consecutive batches within the same container without the need for addition of more brain derived prions. In a LS-PMCA cup container labeled as “RML Bioreactor”, 5 mL of wild-type mouse BH and 5 µL of RML BH were mixed with 100 beads and subjected to PMCA. After amplification, the LS-PMCA product was removed as much as possible by pipetting, stored in a separate container, and designated as “LS-PMCA RML Batch 1”. The RML Bioreactor, without any visible PMCA product to the naked eye, was filled with another 5 mL of wild-type mouse BH without additional RML seed, then subjected to LS-PMCA to generate a second batch of RML prion. This process can be repeated to produce subsequent batches of prions (Figure 5A) and we were able to perpetuate RML prion production in the same RML Bioreactor that requires only a one-time initial minute amount of RML seed (Figure 5B).

Next, we set up 4 RML Bioreactors and, after collecting the first batches of LS-PMCA RML, we stored these Bioreactors at different temperatures for 1 month, namely room temperature, 4°C, −20°C, and −80°C. Afterwards, the 4 Bioreactors were reactivated by adding only substrates and subjected to PMCA. All 4 Bioreactors retained their amplification capacity as RML prions were successfully produced in these cups (Figure 5C). We further showed that washing the Bioreactor with 5 mL PBS between batches did not affect the amplification efficiency (Supplementary Figure S3). This result is likely due to the propensity of prions to adhere to surfaces of various materials (Pritzkow et al., 2018). Storage of the bioreactor for a long time may result in contamination with micro-organisms. Thus, we tested whether washing the container with Aquaguard solution to prevent bacterial and/or fungal growth would affect prion production. After being left at room temperature for 2 weeks, the Aquaguard-washed Bioreactor could still be reactivated by adding just substrates (Figure 5D). Our results show a convenient way to produce large amounts of infectious prions in a container that can be re-utilized indefinitely.

## Discussion

Prions are a unique class of infectious agents composed exclusively of a misfolded and aggregated form of the prion protein that can self-propagate and transfer from individual to individual in the absence of living cells or genetic material (Prusiner, 1998; Soto, 2011). Albeit rare in humans, prion diseases in animals have caused substantial economic and public health problems, such as the transmission of BSE into humans producing vCJD and the uncontrollable spreading of CWD affecting cervids in North America and Europe. Despite substantial advances in prion research, we still do not understand completely the molecular and structural mechanisms implicated in prion replication. The study of traditional infectious diseases in the lab typically depends on the availability of efficient and rapid systems to propagate them *in vitro*. For conventional infectious agents, such as bacteria or viruses, usually cell cultures are used in a bioreactor format. However, since prions do not contain nucleic acids they cannot be propagated in the traditional way. Prion diseases are usually studied using animal models, particularly transgenic mice expressing the prion protein for different species (Moreno and Telling, 2017). Although very informative, such bioassays generally demand costly long-term animal housing and care, plus the ethical concerns associated with research in animals. To understand prion transmission, we invented a technology to propagate prions *in vitro* in an accelerated and efficient manner. This technology named PMCA has been demonstrated as a time- and cost-efficient prion propagation platform, which takes place in test tubes and amplifies prions by millions or even billions of times within days (Castilla et al., 2005a; Morales et al., 2012). More importantly, PMCA propagation preserves the strain properties of the original seed prions and reproduces complex biological properties of prions such as strain maturation (Castilla et al., 2008b; Castilla et al., 2008a; Green et al., 2008) and species barrier transmission (Castilla et al., 2008a). Therefore, PMCA presents a valid substitute for laborious and expensive animal experiments with regard to prion production. However, standard PMCA takes place in a reaction volume of 100 µL in PCR tubes and thus produces a relatively small amount of prions. Considering the principle of PMCA involving cyclic sonication and incubation of prion seeds and substrates, propagation of prions could occur in principle independent of the reaction volume or vessel, as long as PMCA conditions are satisfied. In this study, we implemented, developed and validated an optimized PMCA procedure to produce prions in large scale. LS-PMCA enables to amplify prions in a different reaction container with a much larger reaction volume of material, which results in the production of 1000-time larger amounts of infectious prions as compared with standard PMCA. LS-PMCA readily amplifies prions as efficiently as the standard PMCA assay (Supplementary Figure S1A), even though the LS-PMCA containers have a much thicker wall compared to PCR tubes. The animal assay results further confirmed that LS-PMCA amplifies prions without altering the strain properties (Figure 2). Similar results were observed when the second-generation LS-PMCA vessels were tested, including a more impressive 100 mL reaction (Figure 3), further supporting that LS-PMCA is an efficient way to produce *bona fide* prions in large quantity. Based on comparison by western blots and infectivity bioassay, we estimate that the concentration of PrP^Sc^ produced by LS-PMCA is equivalent to that present in a 10% brain homogenate from an infected mouse at the terminal stage of the disease, but in a much larger volume of material. In our estimation the amount of prions produced in 1d by LS-PMCA using a 100 ml volume is equivalent to that present in 25 mouse brains at the terminal stage of the disease. Since we need to wait a minimum of 150d to produce disease by infection of mice, performing LS-PMCA for this amount of time will produce an amount of prions equivalent to 3750 infected mouse brains.

One of the potential applications of producing large amounts of prions is to facilitate structural studies. The quality and quantity of a protein of interest are key factors for high-resolution structural studies. Despite great advances in prion structural research (Diaz-Espinoza and Soto, 2012), there is not yet sufficient high-resolution structural information of infectious prions, mainly due to either high heterogeneity or low infectious titer of prion fibrils produced *in vitro* from recombinant protein. A couple of recent seminal cryo-electron microscopy (cryo-EM) studies showed that the PK-resistant cores of brain-derived hamster 263K (Kraus et al., 2021) and mouse RML (Manka et al., 2022) prions have a parallel in register inter-molecular beta-sheet structure (PIRIBS). These pioneer studies provided valuable data to understand prion transmissibility and strain variation, and will facilitate the rational design of prion inhibitors. However, both 263K and RML are experimental prion strains that have been passaged and cloned for decades in the lab, which could lead to adaptation and selection of the most stable strain. Thus, it will be interesting to compare these structures with those of natural, first generation prions from relevant species including cattle, cervids and humans. In this sense, two recent articles reported the atomic resolution structure of small fragments of human prions implicated in rare genetic diseases (Hallinan et al., 2022; Li et al., 2022). These studies add substantial value to understand the structure of natural prions, but the structures elucidated correspond to small fragments of the prion protein, which are not readily infectious.

PMCA has also been used to generate prions using recombinant PrP as substrates (Kim et al., 2010; Makarava et al., 2010; Wang et al., 2010; Fernández-Borges et al., 2018; Eraña et al., 2019). However, in most cases recPrions generated exhibit lower infectivity or did not maintain strain properties (Schmidt et al., 2015; Fernández-Borges et al., 2018; Eraña et al., 2019). The LS-PMCA procedure presented in this study uses brain homogenate as substrate, which likely facilitate the faithful replication of prion structures. Firstly, the amino acid sequences and post-translational modifications of PrP substrate in the brain homogenates match 100% the sequences of the seed PrP. Secondly, the brain homogenate substrates likely contain the endogenous co-factors, which have been proposed to be essential for propagating prions and maintaining their strain properties (Soto, 2011). Taken together, akin to conventional PMCA, LS-PMCA is capable of faithfully amplifying prions from various species. Nevertheless, it will be advantageous to use recPrP and purified components for prion replication in our bioreactor. We are currently working on this and indeed recently identified conditions to replicate recPrions that display high infectivity while maintaining strain properties (Wang and Soto, unpublished observations).

Aside from differences in reaction volume and container size, another major difference from standard PMCA is that LS-PMCA has been developed into a Bioreactor format, which provides consistent and robust amplifications under industry-mimicking conditions. A specific LS-PMCA Bioreactor for a certain prion only requires an initial minute amount of prion seed. Then batches of the same prion can be produced continuously in the same vessel with no further need of extra seeding material, which often comes in a limited amount. The LS-PMCA Bioreactor can be washed either with PBS or Aquaguard before being stored at either room or lower temperatures and none of these conditions would impair the production of the prion of interest when the bioreactor is reactivated. This LS-PMCA Bioreactor format presents a remarkably convenient and efficient method for large production of valuable prion strains for biological and structural studies, such as the rare novel Norwegian CWD that was found in a single wild red deer (Figure 4). Moreover, the original prion seeds can be amplified efficiently within just 24 h, which, considering multiple prion sub-strains might co-exist in the same host of natural prion cases (Collinge and Clarke, 2007), diminishes the possibility of strain selection and/or adaptation. Since standard PMCA and LS-PMCA produce infectious material, it is necessary to work with these materials under appropriate biosafety facilities, by well-trained investigators and employ strict measures to avoid infection and contamination. The larger amount of material produced by LS-PMCA compared with conventional PMCA necessitates stringent biosafety procedures and periodic decontamination of equipment and facilities.

Interestingly, we and other investigators have expanded the principles behind PMCA to amplify and detect other prion-like misfolded proteins (e.g., amyloid-beta, tau, alpha-synuclein) implicated in highly prevalent neurodegenerative diseases, including Alzheimer’s, Parkinson’s disease and dementia with Lewy bodies (Salvadores et al., 2014; Fairfoul et al., 2016; Shahnawaz et al., 2017; Metrick et al., 2020; Saijo et al., 2020; Siderowf et al., 2023). These seed amplification assays (SAA) follow the same principles and share many similarities with assays to amplify prions, including PMCA and RT-QuIC (Concha-Marambio et al., 2023). Thus, it is conceivable that the LS-PMCA technology might be expanded to produce large amounts of misfolded aggregates composed of diverse proteins for biological and structural studies.

In summary, in this study we have developed a PMCA-based large-scale production bioreactor system, which is primed to produce a massive amount of prions that preserve the distinct strain features of each prion seed. This system might be very useful to produce large quantities of *bona fide* prions for biological and structural studies, as well as to develop novel strategies for therapeutic intervention, and at the same time to limit the need for the use of large number of animals, which can be costly and time consuming, as well as carry ethical concerns.

## Data Availability

The original contributions presented in the study are included in the article/Supplementary Material, further inquiries can be directed to the corresponding author.
