# Supplementary material for: Development of a methodology for large-scale production of prions for biological and structural studies
Source: Front Mol Biosci. 2023 Aug 10;10:1184029. doi: 10.3389/fmolb.2023.1184029 (PMC10449461; doi:10.3389/fmolb.2023.1184029)

<u>Supplementary Table 1</u>. Incubation times of mice inoculated with material generated by LS-PMCA as well as positive and negative controls.

Animal Group	Survival times<br>Days post-inoculation (DPI)	Incubation period<br>Average ± SEM (DPI)
RML 10% brain homogenate	**170, 167, 170, 175, 182**	**167 ± 2.6**
RML generated by PMCA	**166, 175, 175, 176, 176**	**166 ± 1.9**
RML generated by LS-PMCA	**171, 176, 185, 186, 190**	**171 ± 3.5**
RML 10^{-19} dilution	**300, 500, 500, 500, 500**	**-**
Unseeded PMCA product	**300, 454, 498, 500, 500**	**-**
$Prnp^{-/-}$ PMCA product	**300, 401, 500, 500, 500**	**-**

Supplementary figures

Supplementary figure 1: **A.** The conversion efficiency of the first-generation LS-PMCA was compared to that of the standard PMCA. **B.** To estimate the amount of PrPC converted to PrP^{Sc} by LS-PMCA, 10 μL of RML prion obtained by LS-PMCA was digested with PK and compared to 10 μL of undigested normal mouse BH (N).

Supplementary figure 2: Hamster adapted TME prion strain Hyper was amplified by LS-PMCA. A cup was loaded with 5 mL of normal hamster BH and seeded with 5 μL of HY BH (final dilution of 10^{-4} in the reaction). After 24 hours of programmed sonication (1 round of LS-PMCA), 5 μL of the reaction was used to seed a new cup containing fresh normal hamster BH. Hence, the brain-derived HY prion was diluted additional 1,000 times (10^{-7} after the 2nd round of PMCA). Six rounds were performed, reaching a final dilution of the brain-derived RML prion to 10^{-19}. Samples before (top panel) and after (bottom panel) LS-PMCA are shown

Supplementary figure 3: To study the effect of washing the bioreactor between LS-PMCA rounds, RML prions were subjected to one round of LS-PMCA as described in figure 3B. The amplified product was removed, the bioreactor was extensively washed with PBS and used to amplify the 2nd batch of RML prion.

Supplementary Figure 1

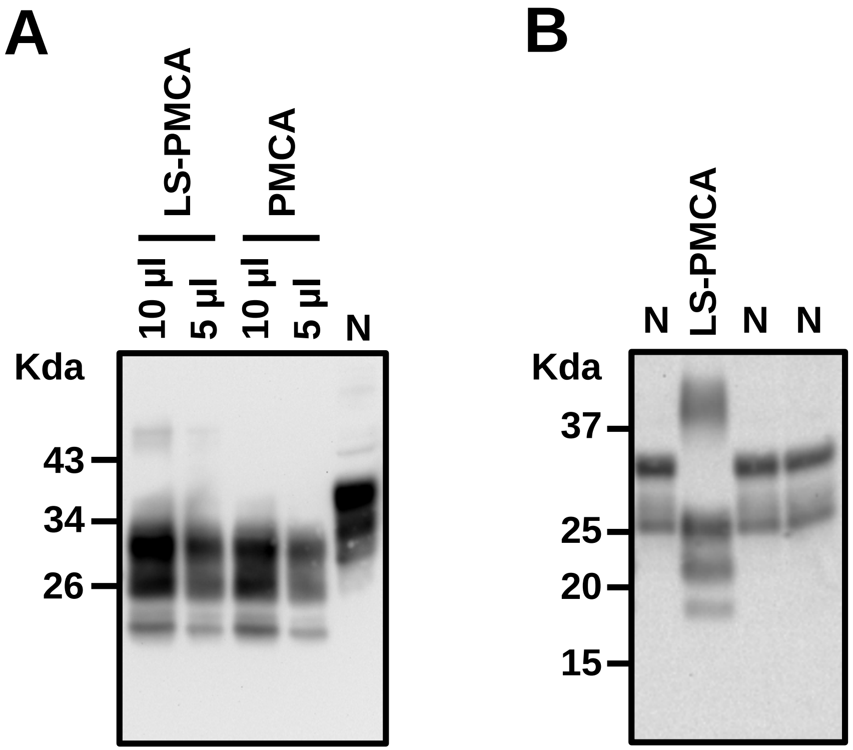

Supplementary Figure 2

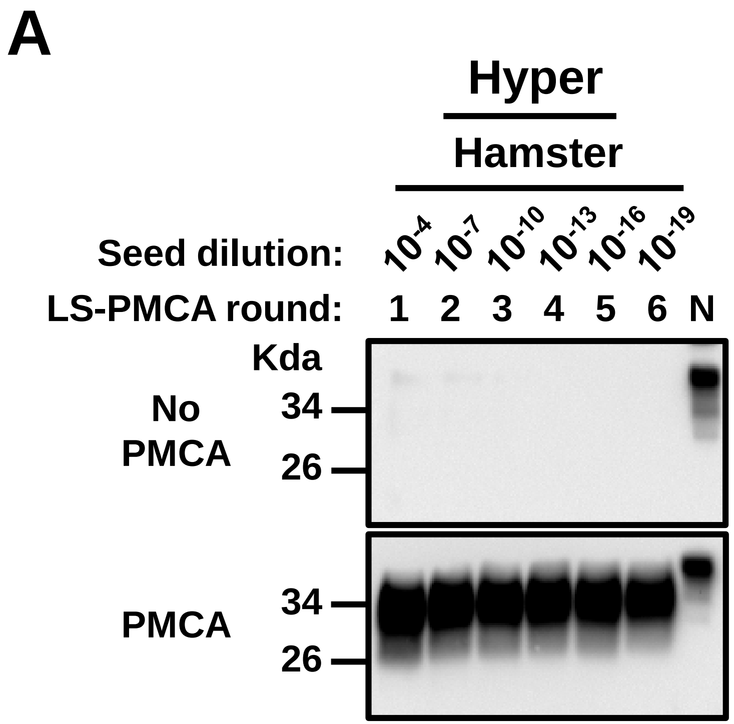

Supplementary Figure 3

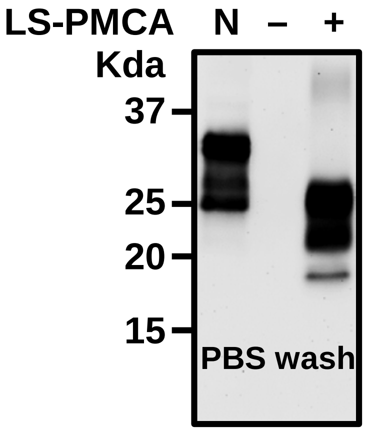

Supplement: Supplementary file 1 [file DataSheet2.PDF]
