# Supplementary material for: Development of a methodology for large-scale production of prions for biological and structural studies
Source: Front Mol Biosci. 2023 Aug 10;10:1184029. doi: 10.3389/fmolb.2023.1184029 (PMC10449461; doi:10.3389/fmolb.2023.1184029)

Figure 1

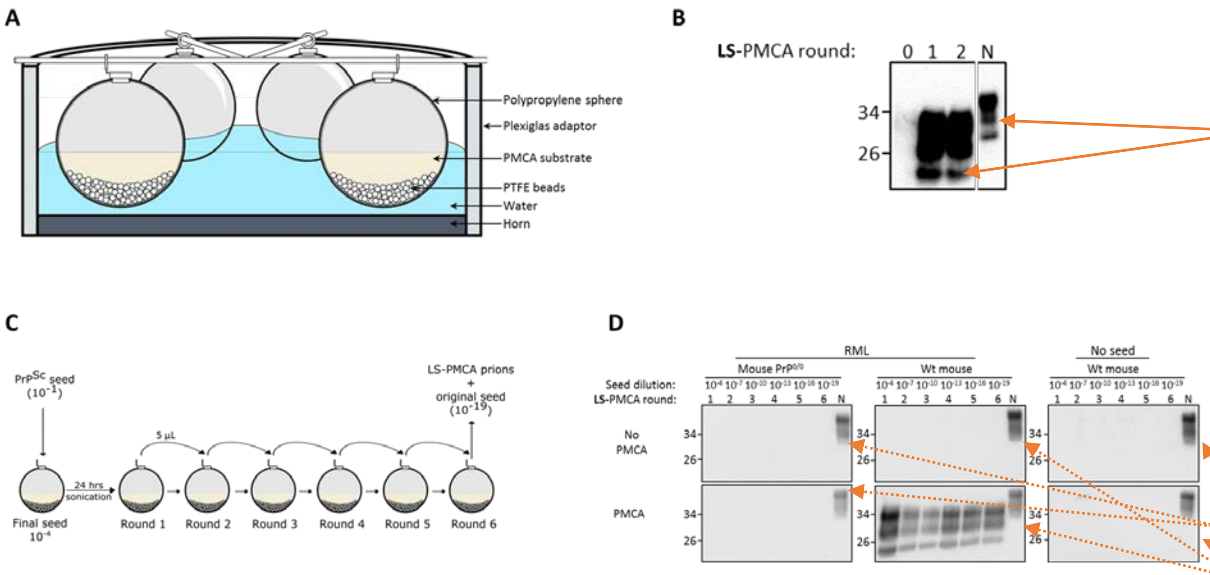

lconchamarambio 2016-08-29 20hr 35min(600)

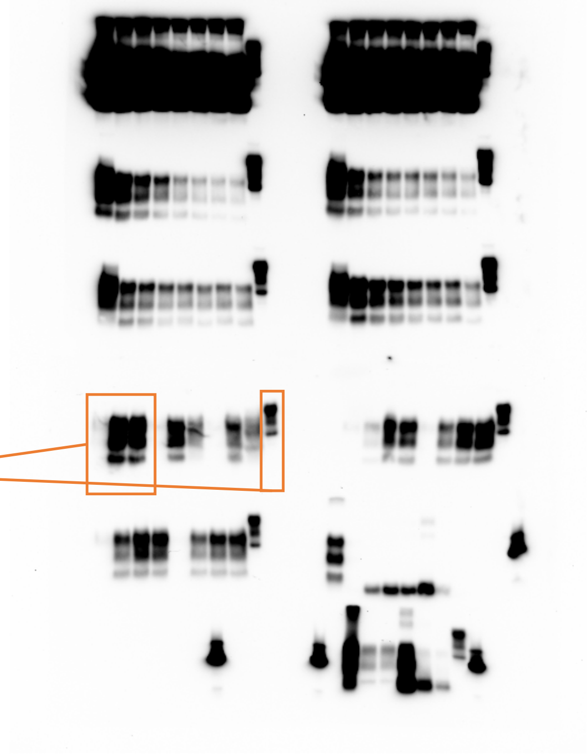

lconchamarambio 2015-03-17 12hr 09min(90)

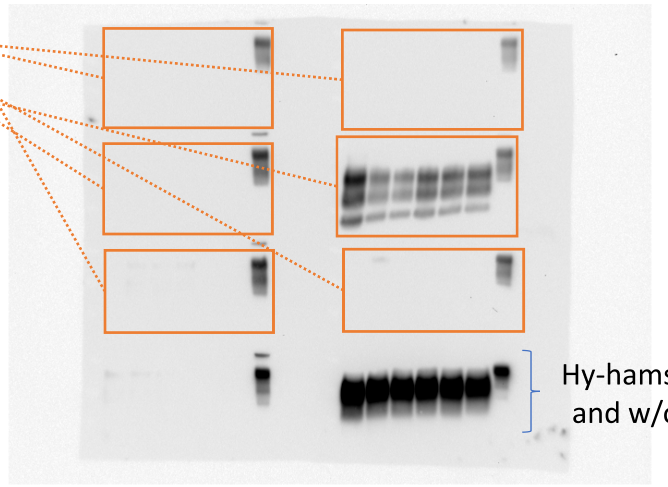

Hy-hamster with and w/o PMCA

Figure 2

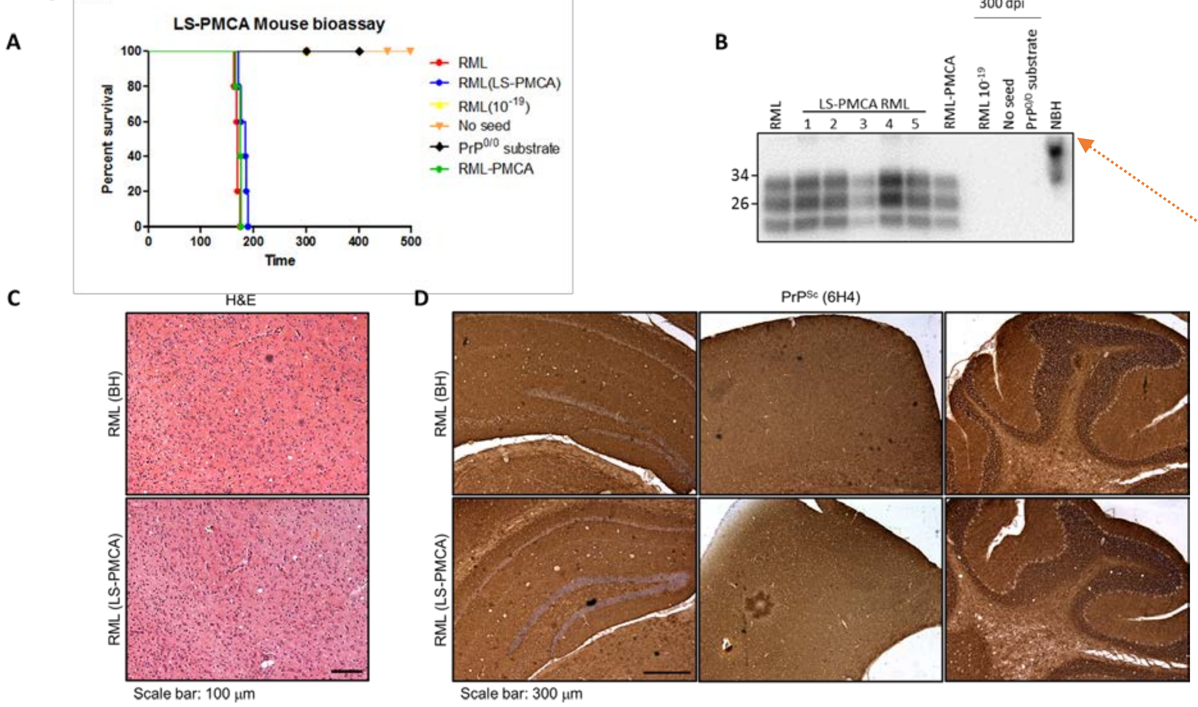

lconchamarambio 2016-07-25 18hr 38min(60)

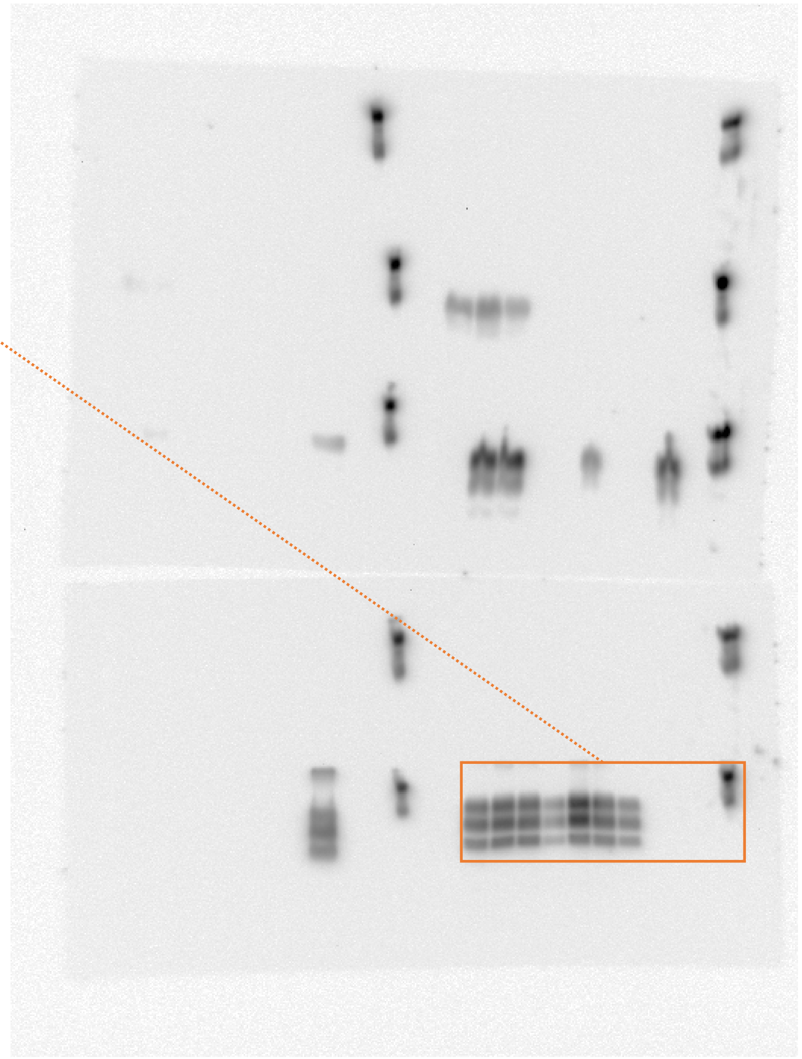

Figure 3

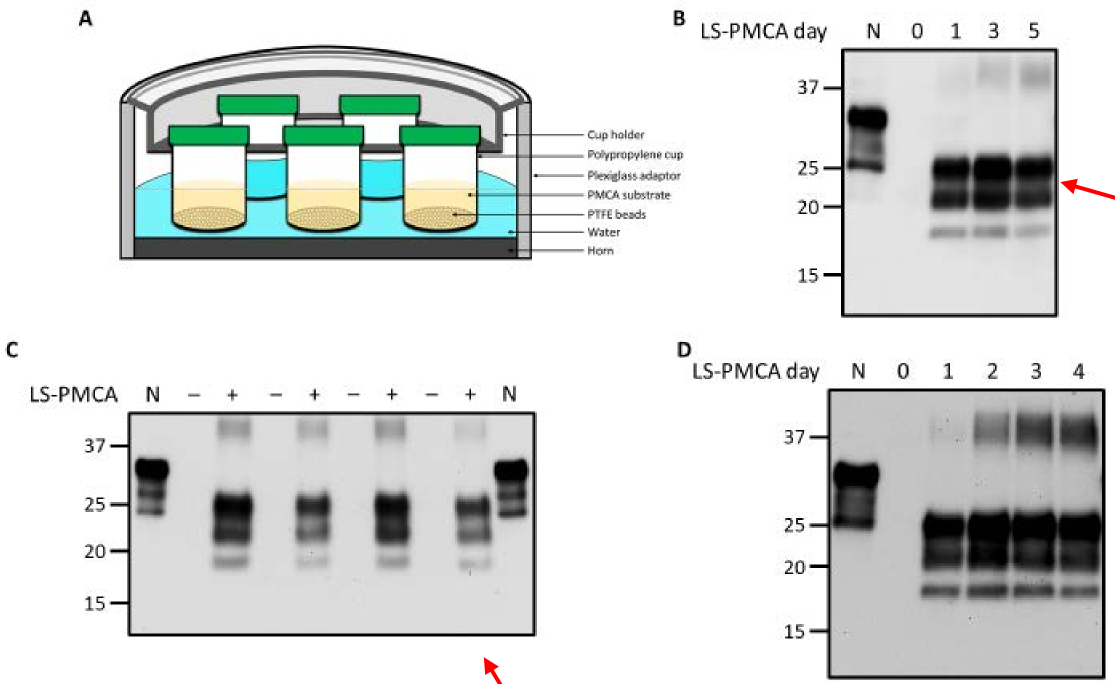

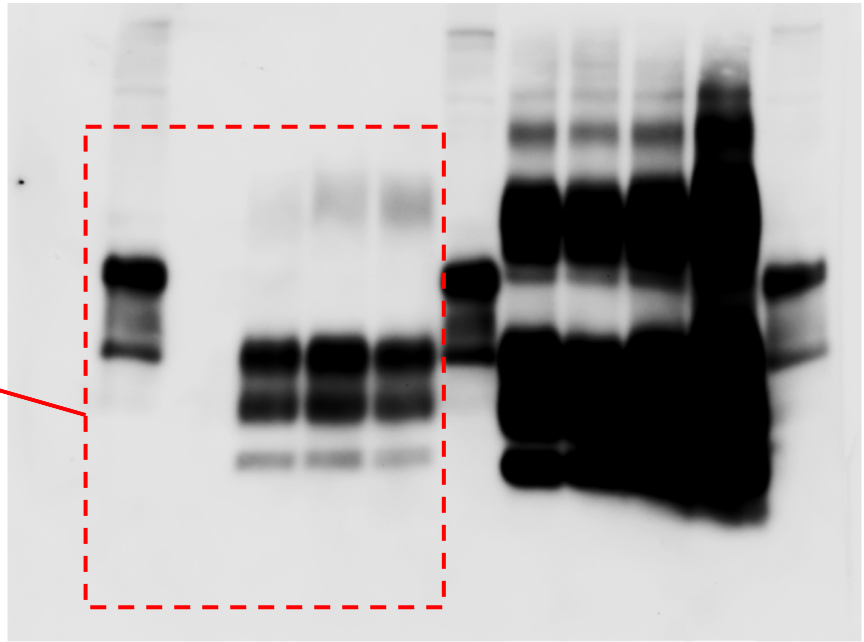

LS-RML Cup8 B1D0 1 3 5, 1%, 2%, 5%, 10% RML BH fwang7 2020-11-20 17hr 46min v10

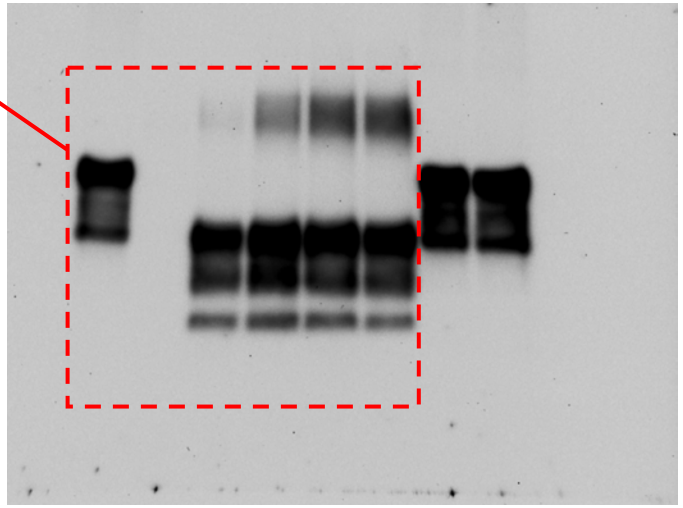

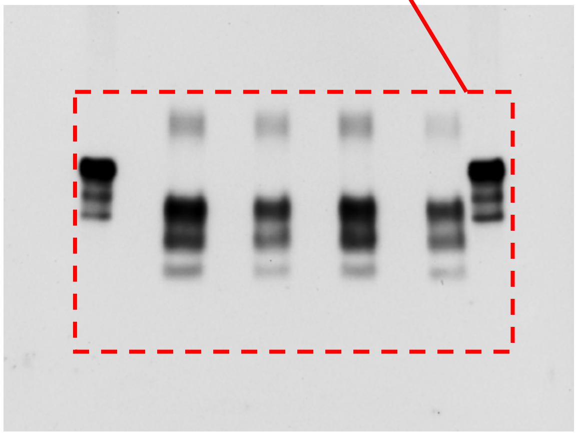

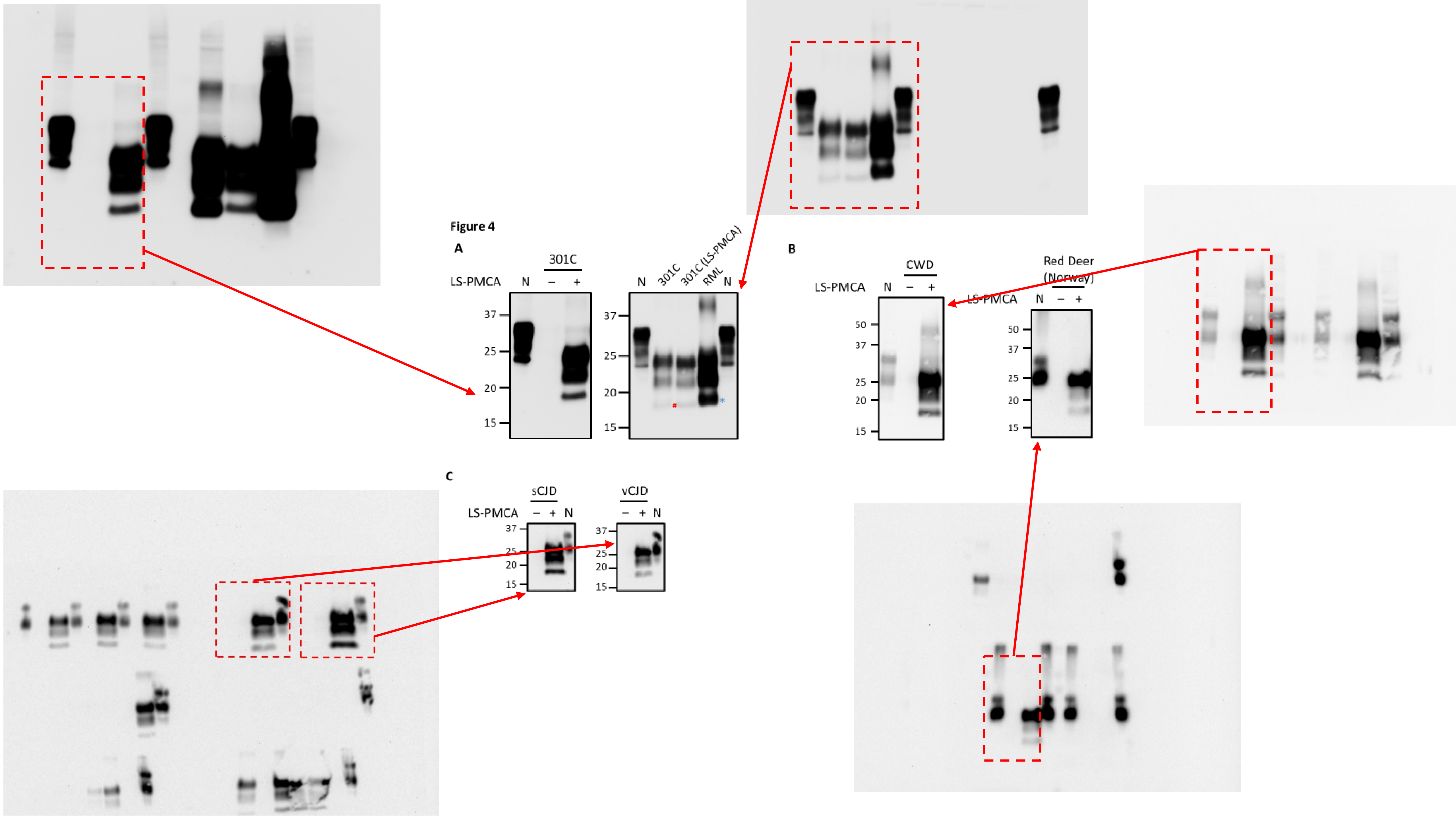
Figure 4
A
301C
LS-PMCA
N
–
+
37
25
20
15
N
301C
301C (LS-PMCA)
RML
N
37
25
20
15
#
*
B
CWD
LS-PMCA
N
–
+
50
37
25
20
15
Red Deer (Norway)
LS-PMCA
N
–
+
50
37
25
20
15
C
sCJD
LS-PMCA
–
+
N
37
25
20
15
vCJD
–
+
N
37
25
20
15

Figure 5

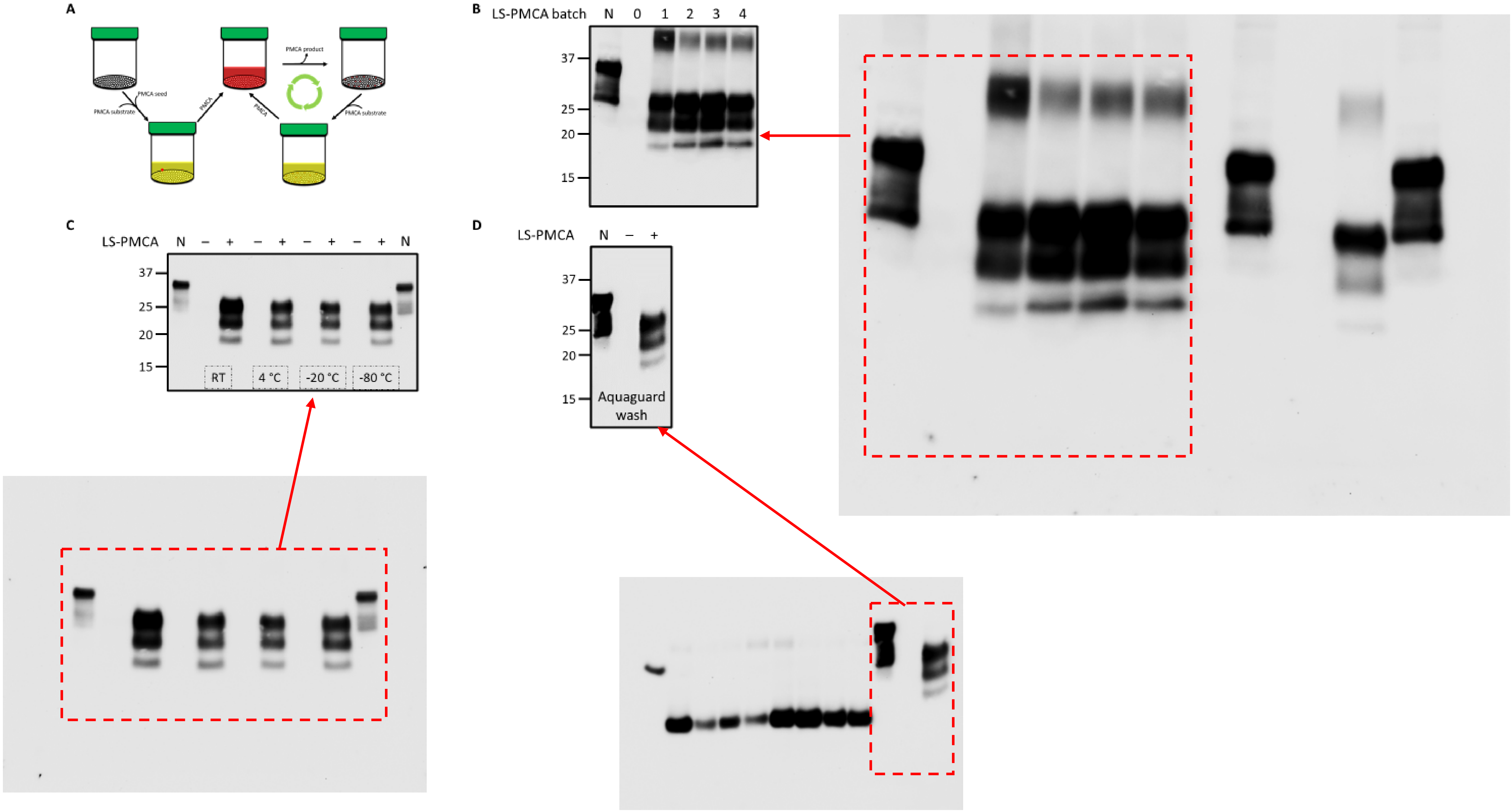
A
PMCA seed
PMCA substrate
PMCA
PMCA product
PMCA substrate
B
LS-PMCA batch
N
0
1
2
3
4
37
25
20
15
C
LS-PMCA
N
–
+
–
+
–
+
–
+
N
37
25
20
15
RT
4 °C
-20 °C
-80 °C
D
LS-PMCA
N
–
+
37
25
20
15
Aquaguard
wash

Supplementary Figure 1

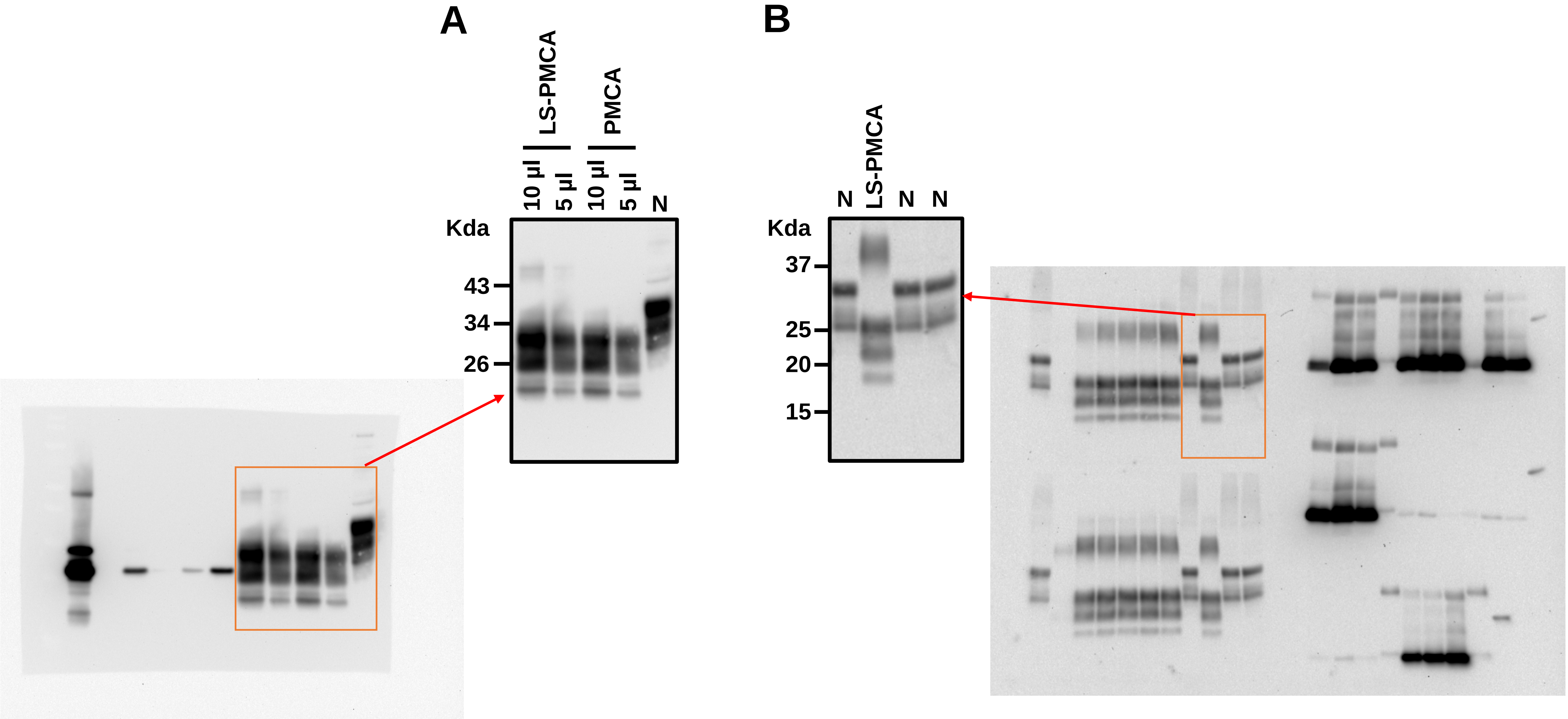

Supplementary figure 2

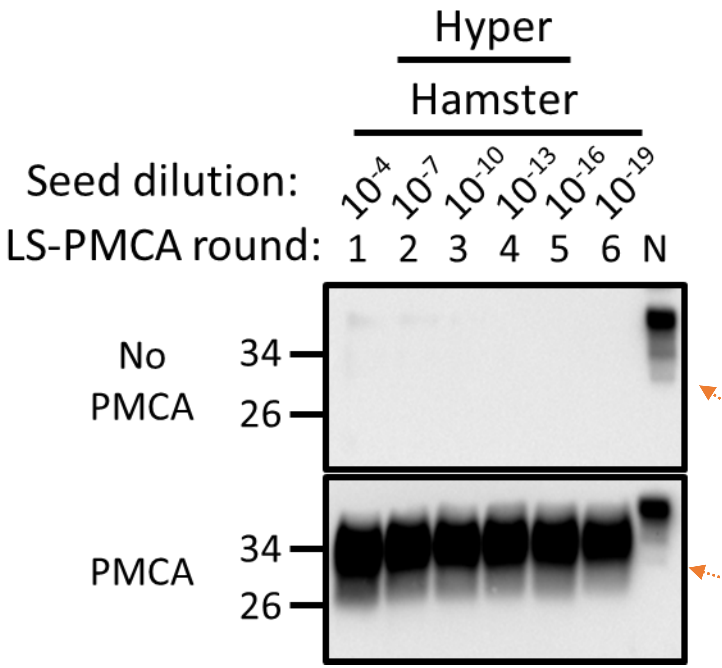

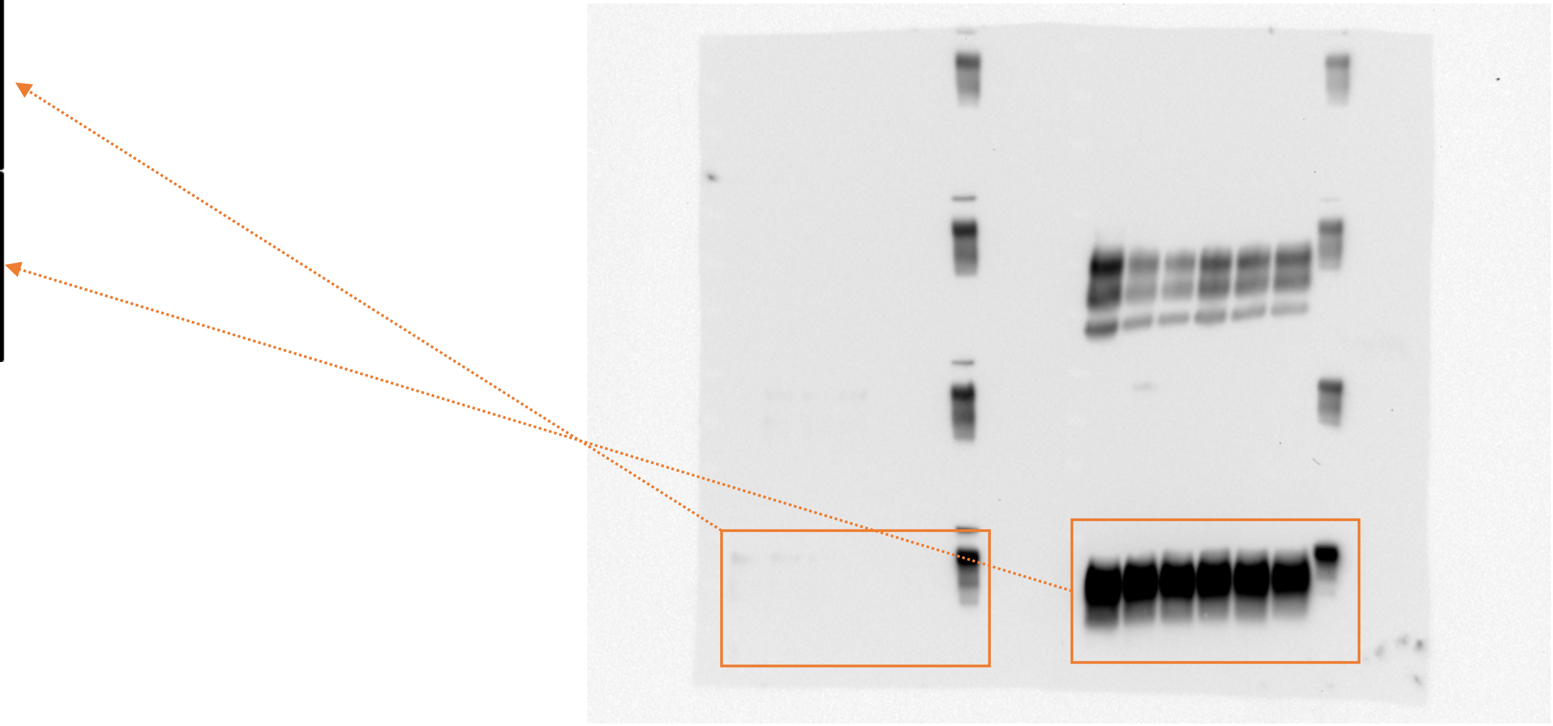

Supplementary figure 3

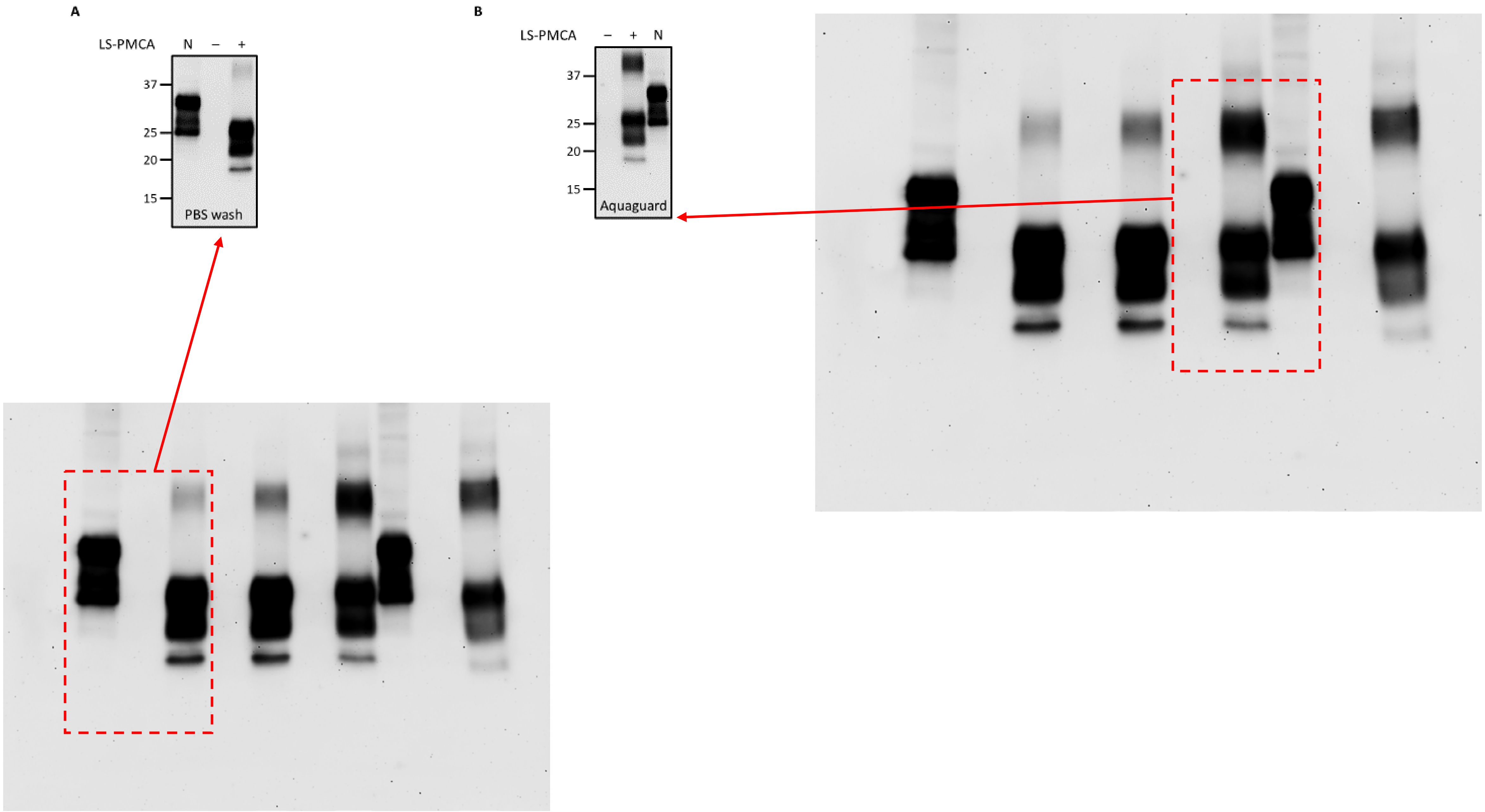

Supplement: Supplementary file 2 [file DataSheet1.PDF]
